# Molecular Detection and Phylogenetic Analyses of Diverse *Bartonella* Species in Bat Ectoparasites Collected from Yunnan Province, China

**DOI:** 10.3390/pathogens11111283

**Published:** 2022-11-01

**Authors:** Guopeng Kuang, Jing Zhang, Weihong Yang, Hong Pan, Xi Han, Lifen Yang, Juan Wang, Tian Yang, Zhizhong Song, Yun Feng, Guodong Liang

**Affiliations:** 1 Yunnan Provincial Key Laboratory for Zoonosis Control and Prevention, Yunnan Institute of Endemic Disease Control and Prevention, Dali 671000, China; 2 School of Public Health, Dali University, Dali 671000, China; 3 Yunnan Center for Disease Control and Prevention, Kunming 650100, China; 4 College of Global Change and Earth System Science, Beijing Normal University, Beijing 100875, China; 5 State Key Laboratory of Infectious Disease Prevention and Control, National Institute for Viral Disease Control and Prevention, Chinese Center for Disease Control and Prevention, Beijing 100052, China

**Keywords:** *Bartonella*, bat, ectoparasite, phylogeny, vector potential, host switch

## Abstract

*Bartonella* species has been validated as blood-borne bacteria in mammals and has a substantial opportunity to be harbored by a variety of hematophagous arthropod vectors. Bats, along with their ectoparasites, are recognized worldwide as one of the natural reservoir hosts for these bacteria. However, there have been few investigations of *Bartonella* bacteria toward a broad range of obligated bat ectoparasites in China. Here, molecular detection of *Bartonella* species was performed to survey the infection among bat ectoparasites and follow-up phylogenetic analyses to further characterize the evolutionary relationships of the genus. A total of 434 bat ectoparasites involving four types of arthropods, namely, bat mites, bat tick, bat fleas, and bat flies (further divided into traditionally fly-like bat flies and wingless bat flies) were collected in 10 trapping sites in Yunnan Province, southwestern China. *Bartonella* was detected by PCR amplification and sequencing through four gene target fragments (*gltA*, *ftsZ*, *rpoB*, and ITS). Accordingly, diverse *Bartonella* species were discovered, including both the validated species and the novel genotypes, which were characterized into several geographical regions with high prevalence. Phylogenetic analyses based on *gltA* and multi-locus concatenated sequences both demonstrated strong phylogeny–trait associations of *Bartonella* species from bats and their parasitic arthropods, suggesting the occurrence of host switches and emphasizing the potential connecting vector role of these ectoparasites. Nevertheless, the maintenance and transmission of *Bartonella* in both bat and hemoparasite populations have not been fully understood, as well as the risk of spillage to humans, which warrants in-depth experimental studies focusing on these mammals and their ectoparasites.

## 1. Introduction

The genus *Bartonella* is a clade of Alphaproteobacteria that contains over 45 species of fastidious, facultative intracellular, Gram-negative bacilli that can globally infect mammalian hosts, and transmission between hosts by hematophagous arthropod vectors [1,2]. Moreover, with the respective reservoir hosts constantly increasing, there are also numerous unclassified *Bartonella* species isolated from animal reservoirs that have not yet been fully characterized, and it is very likely that there are more distinct species than those that have been recognized in previous studies [3]. Currently, at least 13 *Bartonella* species are known to be capable of infecting humans and causing a broad spectrum of diseases, including endocarditis, myocarditis, neuroretinitis, meningitis, splenomegaly, lymphadenopathy, and neurologic disorders [4]. Moreover, it has been proposed that any *Bartonella* species found in animals can cause human infection, which emphasizes the zoonotic importance of these bacteria [5].

Previous research reveals that an extensive range of mammals harbor *Bartonella* bacteria, including rodents, insectivores, carnivores, ungulates, and even marine mammals [6]. Among these animals, bats are one group of particular interest for pathogen-associated research due to their astonishing abundance with worldwide contribution and unique capacity for flying [7]. There has been corroborated evidence that bat immune systems are highly tolerant to infections [8], which may be the reason why this mammal is the natural reservoir for plenty of pathogens, including viruses [9,10,11] and bacteria [12,13]. Not surprisingly, *Bartonella* infections in bats are distributed globally, showing great diversity, with many new strains/genotypes constantly being discovered [14,15,16,17]. There was evidence that bat-borne *Bartonella* possessed the ability to infect humans and showed pathogenic potential [5,18]. In addition, bats were inferred to be the ancestral hosts of all mammal-associated *Bartonella* and profoundly influenced the early geographic expansion of the genus, playing a crucial role in the evolutionary radiation of these bacteria [19].

The experimental studies of blood-sucking arthropods demonstrated that sand flies, louse, fleas, and ticks are competent vectors for transmitting *Bartonella*, and biting flies, mites, and midges also could serve as potential vectors [20,21,22,23]. As it happens, almost all types of these arthropods can parasitize on the surface of bats and be found to have cross-infection with numerous pathogens [24,25]. Diverse *Bartonella* species and genotypes were identified from bat ectoparasites worldwide [26,27], such as bat flies in China [16], the USA [27,28], Western Africa [29], Madagascar [30], Zambia [31], Korea [32], and Malaysia [33]; bat mites in China [16] and Poland [34]; bat ticks in French Guiana [35], Hungary, and Romania [36]; and bat fleas in the USA [37] and Finland [38]. Comparing the phylogenies of *Bartonella* associated with bats and their blood-feeding ectoparasites supports the previous idea that these arthropods serve as natural reservoirs and potential connecting vectors for the bacteria [27]. It is no doubt that more novel strains/genotypes will be identified from bats and their ectoparasites with the increasing amount of research, which will consummate the understanding of *Bartonella* ecology and evolution. However, the maintenance and transmission of *Bartonella* in bat and hemoparasite populations have not been fully understood, requiring more in-depth studies focusing on vector competence.

As an emerging zoonotic pathogen, *Bartonella* species have been identified in bats and their arthropod ectoparasites worldwide. However, previous research on bats in China was lacking [16]. Accordingly, this study aims to investigate *Bartonella* infection towards a broad range of obligated bat ectoparasites in 10 regions of Yunnan Province in China, where various and highly divergent viruses have been discovered in these specimens [25]. These data will provide a more solid foundation for subsequent studies about the co-evolution and host switches for the genus *Bartonella* in bats and their hemoparasite populations in China and even the world.

## 2. Results

### 2.1. Bat Ectoparasites Sampling, Mixing, and Species Identification

**Figure 1:**
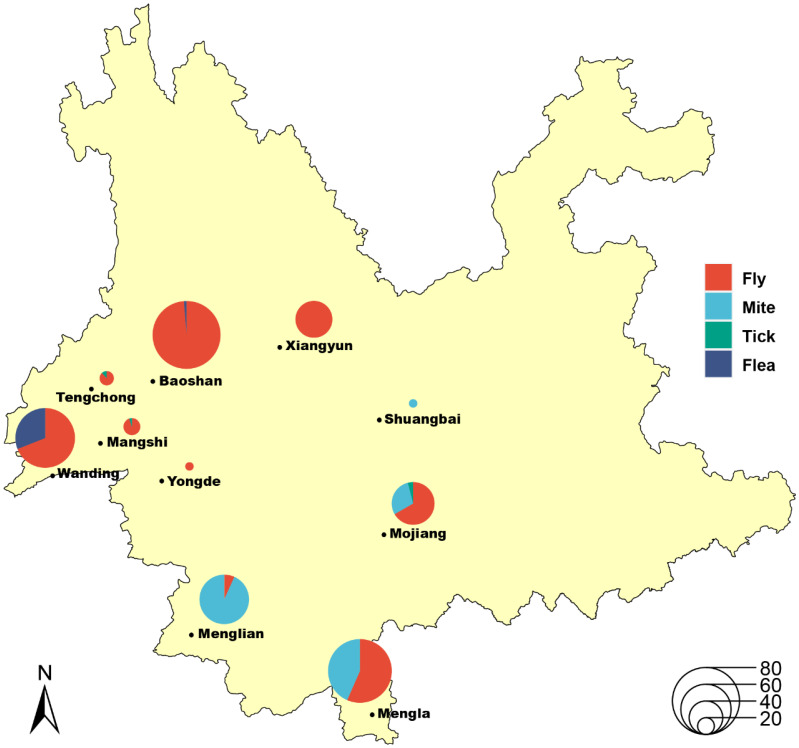
Geographical distribution of ectoparasites in Yunnan, China. The map and pie charts are shaped by using ArcMap 10.8 and RStudio, respectively. The different colors indicate different types of ectoparasites, including bat flies (red), bat mites (cyan), bat ticks (green), and bat fleas (blue). The sizes of the circles represent the number of ectoparasites. The number of each type of ectoparasite was counted, and pie charts illustrate their relative proportions in each trapping site.

A total of 434 bat ectoparasites were collected from 10 cities/counties in Yunnan Province of China between 2012 and 2014, and initial identification was according to the morphological traits—including 295 bat flies, 113 bat mites, 21 bat fleas, and 5 bat ticks (Figure 1, and details are shown in Table S5). All samples were mixed into 40 pools on the basis of the information regarding morphological identification, collected date, and location, and the subsequent species identification was confirmed by sequencing and analyzing the cytochrome c oxidase (*COI*) gene for each pool. The comparison of obtained *COI* sequences in the BOLD database and GenBank showed that there are 28 pools of 295 flies belonging to the superfamily *Hippoboscoidea* (25 pools of 284 wingless bat flies belonging to the family *Nycteribiidae* and 3 pools of 11 traditionally fly-like bat flies belong to the family *Streblidae*), 6 pools of 113 bat mites belonging to the family *Spinturnicidae*, 3 pools of 21 bat fleas belong to the family *Ischnopsyllidae*, and 3 pools of 5 bat ticks belonging to the family *Ixodidae* (Table 1, and phylogenies are shown in Figure S1). The result of molecular biology methods based on *COI* sequences was consistent with initial morphological identification and a previous study that identified through the use of the meta-transcriptomic approach [25].

### 2.2. Bartonella Detection

For all mixed ectoparasite pools, four genes (*gltA*, *ftsZ*, *rpoB* and ITS) were used to detect *Bartonella*. A total of 20 pools involving four types of bat ectoparasites collected in the current study were found to harbor *Bartonella* by *gltA* screening and the following detection of targeted *ftsZ* genes: 2 (66.7%) of 3 bat flea pools, 1 (33.3%) pool of 3 bat tick pools, 4 (66.7%) of 6 bat mite pools, and 13 (52.0%) pools of 25 wingless bat fly pools were positive to detection, and all three fly-like bat fly pools were negative (Table 1). Several pools were negative to PCR detection in genes *rpoB* and ITS, and therefore, we failed to obtain a part of some novel strains’ targeted gene fragment sequences (Table S1).

The bat ectoparasite populations of all 10 trapping sites in Yunnan province are shown to be widely infected with *Bartonella*, wherein the wingless bat fly showed the highest prevalence ratio of *Bartonella* (eight of nine sites were positive to detection), whereas the traditional bat fly was the lowest (all sites are negative to detection) (Table 1). Moreover, several sites with more than one type of hemoparasite harbored *Bartonella*, such as Wanding Town (bat flea and wingless bat fly) and Mengla County (bat mite and wingless bat fly) (Table 1).

### 2.3. Molecular Biological Characteristics of Newly Discovered Bartonella

#### 2.3.1. Identification of Bartonella

**Figure 2:**
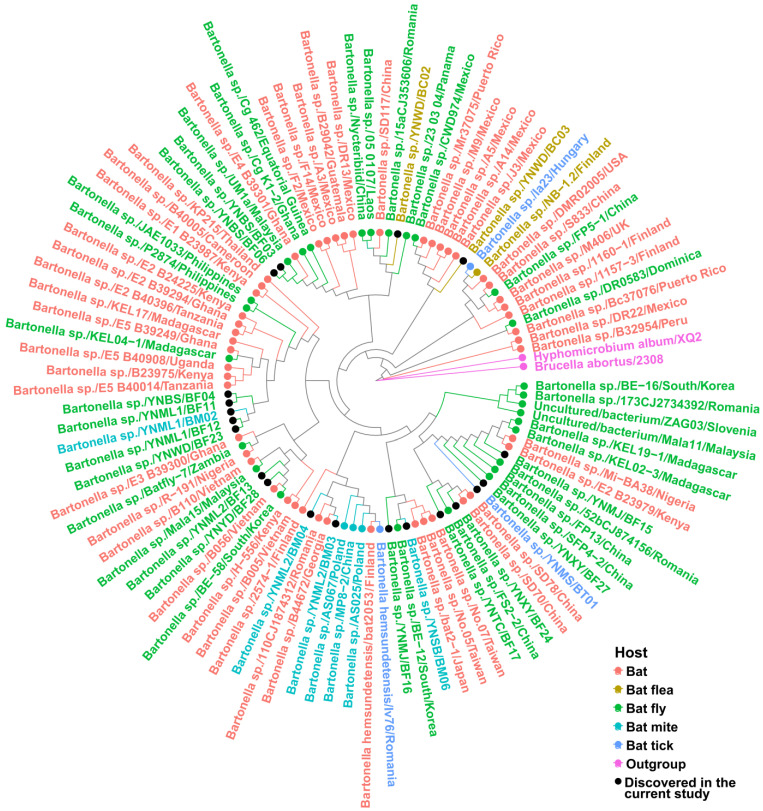
Identification of novel strains according to the phylogenies of *Bartonella* associated with bats and bat ectoparasites based on the gltA gene. The *Bartonella* strains newly identified in the current study are marked with solid black circles within the phylogenetic tree. Host groups are indicated with different colors: bat (red), bat flea (golden), bat fly (green), bat mite (cyan), and bat tick (blue). *Hyphomicrobium album* str. XQ2 and *Brucella abortus* str. 2308 were used for the outgroup and are indicated with pink. The phylogenetic tree was constructed using the maximum likelihood method and visualized by RStudio.

We identified the novel detected strains by nucleotide sequence similarities and phylogenies of the gene *gltA*, the most common target for *Bartonella* detection with good discriminatory power of delimitation. According to the nucleotide BLAST analysis of approximately 380 bp *gltA* fragment sequences in the GenBank, the similarity of those detected from bat ectoparasites in this study with the validated species/strains ranged from 88.42% in the family *Ischnopsyllidae* (YNWD/BC02) to 100% in the family *Spinturnicidae* (YNML2/BM03 and YNML2/BM04) (Table 2). Out of all 20 novel strains, there were 12 strains of novel species, tentatively named *Bartonella* sp., which shared <96% sequence similarity with known species/strains discovered by previous studies, while the other 8 strains were the same as the previously described genotype with >96% identity. Likewise, the phylogeny analysis by the maximum likelihood tree based on the *gltA* sequences of obtained and representative *Bartonella* strains associated with bats and bat ectoparasites put themselves into the same clade with the closest strains to those showing the highest identity (Figure 2).

On the other hand, the clustering traits of *Bartonella* sp. in the current study were neither strongly associated with host family taxonomy nor geographic origin, the same as the strains from bats and their parasites in previous studies (Figure 2). Firstly, the strains from different host arthropods in the same trapping sites closely clustered in the same clade, for instance, the strain YNML1/BF12 from bat flies pool and YNML1/BMo2 from bat mites pool (and shown 98.9% similarity). Secondly, the strains detected in the same type of ectoparasite (bat fly) from Mojiang County, YNMJ/BF15 and YNMJ/BF16, did not cluster in the same clade (86.8% similarity), likewise with the flea-borne strains YNWD/BC02 and YNWD/BC03 from Wanding Town (85.3% similarity).

#### 2.3.2. Phylogenetic Analyses

Three gene (*gltA*, *ftsZ*, and *rpoB*) fragment sequences were concatenated and, together with validated *Bartonella* species/strains that were discovered in nine types of hosts at relevant taxonomic scales of the order level, to infer a more comprehensive phylogeny of this genus (Table S3). Ultimately, an approximately 2017 bp length of concatenated sequences for three loci, 380-bp, 786-bp, and 851-bp of genes *gltA*, *ftsZ*, and *rpoB* partial sequences, respectively. The concatenated sequences and reference strains were used to construct the phylogenetic tree using the maximum likelihood method.

**Figure 3:**
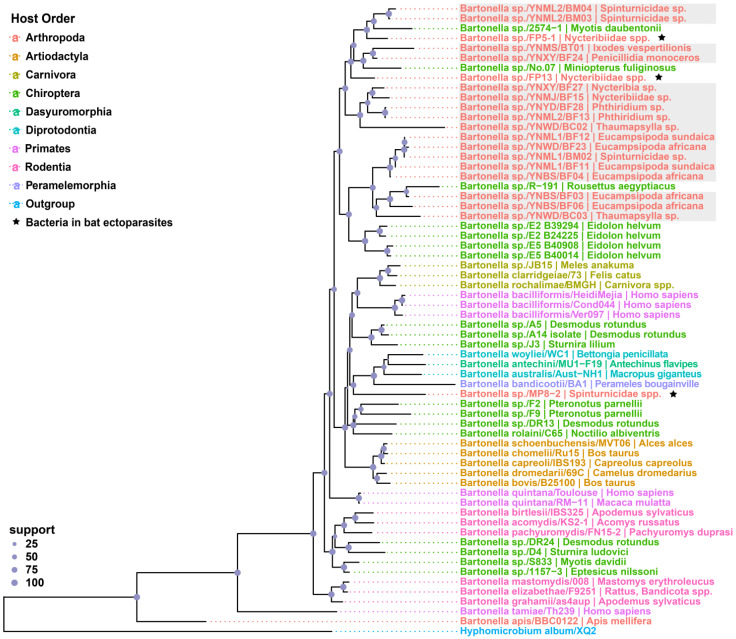
Maximum likelihood tree of the genus *Bartonella* based on three-loci (*gltA*, *ftsZ*, and *rpoB*) using the MLSA approach. The host species of each strain is shown behind the bacteria names, and the different colors indicate different host order: Arthropoda (red), Artiodactyla (orange), Carnivora (golden), Chiroptera (green), Dasyuromorphia (aqua), Diprotodontia (cyan), Primates (pink), Rodentia (rose), and Peramelemorphia (purple). Within the phylogenetic tree, the *Bartonella* strains newly identified here are marked with gray shaded squares, and the strains associated with bat bugs are marked with black pentagrams. *Hyphomicrobium album* strain XQ2 was used for the outgroup and is indicated with blue.

Phylogenetic analysis indicates that clades of *Bartonella* lineages seem broadly host-specific within the host order, except for those associated with bats (Figure 3). Bats and their bug-associated strains formed several clades and external branches to other mammalian orders, which were dispersal distributed across the tree. The tree put all strains of the current study into two close clades of monophyletic groups associated with bats and closely related to the strains, namely, *Bartonella* sp. FP5-1 and FP13, which were detected from bat flies in Jingzhou City, central China.

## 3. Discussion

A total of 434 bat-parasitic arthropods collected from 10 trapping sites in Yunnan were used to perform a molecular investigation of *Bartonella*. Morphological identification and confirmation by analysis based on the cytochrome c oxidase (*COI*) gene revealed five families of bat-specific ectoparasites were contained, namely, *Nycteribiidae* (wingless spider-like bat flies), *Streblidae* (traditionally fly-like bat flies), *Spinturnicidae* (bat mites), *Ischnopsyllidae* (bat fleas), and *Ixodidae* (bat ticks). All bat ticks were identified as *Ixodes vespertilionis* or *Ixodes collaris*, two species of ticks that exclusively parasitize bats [39,40]. All bat mites and bat fleas were unclassified species of *Spinturnicidae* sp. and *Thaumapsylla* sp., respectively. In contrast, diverse bat flies were discovered, including both named species such as *Eucampsipoda africana/sundaica*, *Penicillidia monoceros*, and *Brachytarsina kanoiand*, and the potentially novel species tentatively named *Nycteribiidae* sp., *Phthiridium* sp., *Nycteribia* sp., and *Brachytarsina* sp. (Table S1). Host species information was initially identified according to morphological traits, and all specimens were later mixed on the basis of the above information and further confirmed by sequencing and analyzing the *COI* gene for each pool. Therefore, the accuracy of initial information was highly dependent on experienced field biologists, which was important for the subsequent pooling and gene identification. We believe the combination of morphological characteristics and molecular analysis will better define these bat ectoparasites in the future.

Although diverse *Bartonella* strains/genotypes have been identified in bats and their ectoparasites worldwide [16,19,26,27], including in China [16,41,42], there have been few reports in China. In this study, molecular detection of four gene target fragments (*gltA, ftsZ, rpoB*, and ITS) was performed to survey the infection of *Bartonella* among bat ectoparasites as described above. In sum, 20 mixed pools were positive for detecting the gene *gltA* and *ftsZ*, while 17 and 18 were positive for genes *rpoB* and ITS, respectively. Four types of ectoparasites, namely, bat mites, bat fleas, bat ticks, and wingless bat flies, were discovered to harbor *Bartonella* bacteria. Moreover, the bat ectoparasite populations of all 10 trapping sites were found to be commonly infected with the *Bartonella* bacteria (Table 1), which somehow reflects a high prevalence ratio of these bacteria in Yunnan province. To our knowledge, it is the first time these bacteria have been detected in bat fleas and ticks in China, and there have been few discoveries around the world regarding *Bartonella* infections in these two types of bat-parasitic arthropods.

Due to the *gltA* gene having good discriminatory power of delimitation [43,44], limited genetic sequencing has permitted the tentative identification of *Bartonella*. We propose that the detected strains be considered a new species if a >327 bp *gltA* fragment shares <96.0% sequence similarity with the validated species, whereas those with >96% identity are the same genotype [43]. Accordingly, 12 strains of novel species and 8 strains of validated genotypes were detected, revealing bat ectoparasites infected with a diversity of these bacteria (Table 1), and with many novel genotypes not overlapping with those from bats (Figure 1), suggesting the role of natural reservoirs of *Bartonella* for these arthropods. On the other hand, the close relationship of strains from bat mites and bat flies indicated that ectoparasites serve as the potential connecting vector of *Bartonella* bacteria (Figure 1), which further supports the same idea of the previous study [27]. Nevertheless, there is an essential difference between proven vector competence and vector potential, requiring further experimental vector transmission studies to confirm this.

Previous studies showed that *Bartonella* lineages are broadly host-specific within orders [19,45,46]; however, host-switching seems to occur between “closely related” animals. The maximum likelihood tree demonstrated a strong phylogeny–trait association of *Bartonella* spp. from bats and their parasitic arthropods, forming several complex bat/ectoparasite-associated *Bartonella* lineages (Figure 2 and Figure 3). We suspect the high host specificity and co-feeding behavior of obligated ectoparasites is one of the vital factors [47]. The long history of co-evolution between mammals, parasitic ectoparasites, and harboring bacteria may significantly cause the complex *Bartonella* lineages that cluster genetically similar strains from different host types. Given that diverse bacteria are harbored by bats [12,13], the same event of reservoir spillover to parasitic arthropods and divergent adaption can occur in other lineages of host–bacterial systems. On the other hand, the *Bartonella* associated with bat bugs and bat-borne strains form the external branches to other mammalian orders within the tree (Figure 3), which again confirms the previous view that bats have a deep influence on the radiation of mammal-associated *Bartonella* bacteria [19]. In addition, the previous studies demonstrated that the diversification of mammal-infecting eubartonellae started almost exactly when bats began their evolutionary radiation [19,48,49], also supporting the fact that bats play a crucial role in the genomic evolution of these bacteria. Even though these potentially confounding factors are discovered, in-depth analyses are necessary for understanding the coevolutionary patterns and frequency of host-switching events.

Bats are the natural reservoir for many pathogens [9,10,11,12,13], including *Bartonella* bacteria [19,38,41]. A bat-associated *Bartonella* species, proposed as *Bartonella rousetti* (*Bartonella* sp. strain R-191), was corroborated, potentially being capable of infecting humans by serologic evidence [5]. Although phylogenetic analysis shows strain R-191 closely clustered with the fly-associated strain YNBS/BF03 in this study (Figure 3), whether these ectoparasite-borne *Bartonella* strains hold the same infecting capability still relies on further experimental infection studies. We only focused on the *Bartonella* infection of bat ectoparasites in this study but lacked a detection of their host bats; therefore, an investigation of bat populations in these trapping sites and even wider regions should be taken up to clear the infection situation of these bacteria. Moreover, serology is critical in diagnosing *Bartonella* infections [3], and corresponding surveillance of the local human populations is also needed. More studies are required to elucidate the correlations between exposure routes and the pathogenicity of ectoparasite-borne *Bartonella* sp. in humans.

Overall, molecular detection of *Bartonella* in the bat ectoparasites revealed diverse bacterial species infection, and sequencing of multi-locus and phylogenetic analysis allows for a deeper understanding of host ecologies and latent potential for vectors and the evolutionary traits of these bacteria. However, there are some limitations revealed in this preliminary investigation. Firstly, as previously described, the number of arthropod samples especially bat ticks was limited, except for wingless bat flies (Figure 1) [25]. In addition, a mixed operation of specimens and sampling bias towards ectoparasites complicated the interpretation of relationships between the bacteria and hosts. Secondly, although the specimens were collected alive from the body surface of stuck bats and left in gauze bags for up to 24 h to digest the sucking blood before being transferred to liquid nitrogen, we cannot entirely eliminate the possibility that the bacteria were detected from the blood of bats but have no infection of bat ectoparasites because of the no-cultivation of the organism. Lastly, few bat ectoparasite-associated references are contained for the MLSA analysis because of a large number of sequence gaps (*gltA*, *ftsZ*, or *rpoB*) among the published strains, which limits a complete phylogeny of bacteria from bats and their parasitic arthropods. With increasing sampling and a wider variety of bats and their ectoparasite, we expect additional *Bartonella* strains/species to be identified, and more available data can be analyzed towards ecologies and phylogenies of these bacteria and their hosts.

## 4. Material and Methods

### 4.1. Ethics Statement

The procedures and protocols of sample collection and processing were reviewed and approved by the Medical Ethics Committee of the Yunnan Institute of Endemic Diseases Control and Prevention (20160002). All the experiments were performed with approval by the Biosafety Committee of the Yunnan Institute of Endemic Diseases Control and Prevention.

### 4.2. Sample Collection

Bats were captured using sticky nets in orchards and caves of 10 counties/cities/towns in central and southwest Yunnan Province, including Xiangyun County, Shuangbai County, Baoshan City, Tengchong City, Mangshi City, Wanding Town, Yongde County, Menglian County, Mojiang County, and Mengla County (Figure 1). All visible ectoparasite specimens were collected from the body surface of stuck bats carefully using tweezers, then the bats were set free. The collected bat ectoparasites were placed into tubes with records and transported back to the local laboratory soon as possible; then, they were left in gauze bags for up to 24 h to digest the blood. Initial identification was made using morphological features such as the head, abdomen, wings, and legs under a stereo microscope, and following this, they were sealed, frozen in liquid nitrogen, and transported to our laboratory and kept at −80 °C.

### 4.3. Sample Mixing, Deoxyribonucleic Acid Extraction, and Host Species Identification

The samples were poured into a precooled sterile grinding mortar and washed with 2 mL minimal essential medium (MEM); then, the liquid was discarded and we added 1 mL of MEM containing 10% penicillin–streptomycin solution. All bat ectoparasites were homogenized individually at low temperatures until the disappearance of the tissue mince [50]. The grinding fluid was centrifuged at 18,000 rpm and 4 °C for 30 min. All 434 supernatant fractions were combined into 40 pools on the basis of the information regarding morphological identification, collected date, and location. Accordingly, 284 wingless bat flies were mixed into 25 pools, 11 fly-like bat flies into 3 pools, 113 bat mites into 6 pools, 21 bat fleas into 3 pools, and 5 bat ticks into 3 pools (Table S1). A total volume of 200 μL of mixing homogenate was used to extract DNA using a TIANamp Genomic DNA Kit (Tiangen Biotech, Beijing, China). Subsequent species identification was based on the cytochrome c oxidase subunit I (*COI*) gene. The *COI* gene sequences of all mixing pools were obtained by Sanger sequencing as previously described (Table S4) [50,51], and the sequences were confirmed by comparison against the BOLD database (http://www.boldsystems.org/ (accessed on 7 September 2022)) and NCBI nucleotide database.

### 4.4. Bartonella Detection and Identification

*Bartonella* bacteria were detected by conventional PCR amplification and sequencing of several a variety of genes (*gltA*, *rpoB*, *ftsZ*, and ITS) primer pairs as described previously (Table S4) [52,53,54,55]. The pools were first screened with the partial *gltA* gene because it is the most common target for *Bartonella* detection and identification [43]. On the basis of the initial PCR result, the *gltA*-positive pools were also detected with the genes *ftsZ*, *rpoB*, and ITS to further confirm and characterize the bacteria. The PCR reaction was performed in a 25 μL mixture containing 12.5 μL 2 × DreamTaq Green PCR Master Mix (Thermo Scientific, Lithuania), 1 μL of 10 μM each forward and reverse primer (Sangon Biotech Co., Ltd., Shanghai, China), 8.5 μL nuclease-free water, and 2 μL sample DNA. PCR was performed with one denaturation cycle at 95 °C for 5 min; 40 amplification cycles at 94 °C for 30 s, 50 °C for 60 s, and 72 °C for 90 s; and an additional final extension at 72 °C for 10 min. All PCR products were separated on a 1.2% agarose gel electrophoresis and visualized by E-Gel Imager (Tanon 2500B) with GoldView staining, and those observed bands of the expected size were purified and subsequently sequenced by Sangon Biotech. Sequences were determined and assembled using the SeqMan program implemented in the DNASTAR software package (Lasergene). Assembled sequences were compared to known sequences in GenBank using the Nucleotide BLAST (BLAST + 2.13.0), and identification of the obtained *Bartonella* sp. was according to the homology.

### 4.5. Phylogenetic Analysis

The single locus, especially *gltA*, was used to analyze the phylogenetic resolution between *Bartonella* species and subspecies because this gene has good discriminatory power to delimit genotypes and species of these bacteria [44]. The *gltA* reference sequences, which contained the representative published strains identified from bats and their ectoparasites, were used for the classification of novel strains were collected from GenBank (Table S2). However, previous *Bartonella*-associated studies have shown the limitations of the *gltA* gene in individually resolving phylogenetic relationships because of the occurrence of genetic recombination [56], and therefore, we amplified and sequenced the additional two protein-coding loci (*ftsZ* and *rpoB*) to further characterize the phylogenetic traits using the multi-locus sequence analysis (MLSA) approach [19,53]. In contrast, the gene ITS was not included due to many sequence gaps among the reference strains. The validated *Bartonella* species/strains that were discovered in nine types of hosts at relevant taxonomic scales of the order level were used for MSLA, aiming to characterize the evolution of the bacterium (Table S3). MAFFT was used to align the nucleotide sequences [57], and the terminal sequences were removed manually and then pruned sequences using trimAl [58]. Phylogenetic trees were constructed using PhyML by the maximum likelihood method and a bootstrap value of 1000, with the GTR + G substitution model and SPR tree topology optimization algorithm [59]. The phylogenetic trees were illustrated by using ggtree objects implemented in RStudio.

## 5. Conclusions

Continuous discoveries of *Bartonella* from bat ectoparasites worldwide suggest these arthropods may serve as both natural reservoirs and potential connecting vectors. However, few investigations have focused on these bacteria towards bat-parasitic ectoparasites in China, especially bat fleas and ticks. We surveyed the *Bartonella* infection in a broad range of obligated bat ectoparasites, including bat flies, bat mites, bat fleas, and bat ticks, finding that these arthropod populations widely harbor diverse *Bartonella* species with a high prevalence ratio. Bacterial phylogenies combine the host’s taxonomy, indicating the occurrence of reservoir spillover to bat ectoparasites. Moreover, a strong phylogeny–trait association between *Bartonella* sp. in these blood-sucking arthropods and bat hosts demonstrated the vector potential of ectoparasites. Nevertheless, the molecular epidemiological characteristics of these bacteria still have not been fully understood, which is recommended for follow-up surveillance of both bats and local human populations in this region and in even wider regions of Yunnan Province in China.

## Figures and Tables

**Table 1:** Result of *Bartonella* detection by using PCR in bat ectoparasites.

Ectoparasite Family	Location	Number of Ectoparasites	Number of Mixed Pools	*Bartonella* PCR			
				*gltA*	*ftsZ*	*rpoB*	ITS
*Ischnopsyllidae*	Baoshan	1	1	0	0	0	0
(Bat flea)	Wanding	20	2	2	2	2	1
*Ixodidae*	Mangshi	1	1	1	1	1	1
(Bat tick)	Mojiang	2	1	0	0	0	0
	Tengchong	2	1	0	0	0	0
*Spinturnicidae*	Mengla	33	2	1	1	1	1
(Bat mite)	Menglian	55	2	2	2	2	1
	Mojiang	15	1	0	0	0	0
	Shuangbai	10	1	1	1	0	1
*Streblidae*	Baoshan	1	1	0	0	0	0
(Bat fly)	Mojiang	8	1	0	0	0	0
	Wanding	2	1	0	0	0	0
*Nycteribiidae*	Baoshan	79	6	3	3	3	3
(Wingless bat fly)	Mangshi	19	3	0	0	0	0
	Mengla	43	2	2	2	2	2
	Menglian	4	1	1	1	1	1
	Mojiang	26	2	2	2	1	2
	Tengchong	15	2	1	1	0	1
	Wanding	44	4	1	1	1	1
	Xiangyun	44	4	2	2	2	2
	Yongde	10	1	1	1	1	1

**Table 2:** Result of *gltA* sequence BLAST in GenBank.

NO.	Pools	Classification	Strains	Host Species	BLASTn Hits on Known *Bartonella* (BLAST Nucleotide Identity)
1	YNML1/BM02	*Bartonella* sp.	YNML1/BM02	*Spinturnicidae* sp.	KM030506/*Bartonella* sp./B23975 (93.42%)
2	YNML2/BM03	*Bartonella* sp.	YNML2/BM03	*Spinturnicidae* sp.	MK140370/*Bartonella* sp./B44672 (100%)
3	YNML2/BM04	*Bartonella* sp.	YNML2/BM04	*Spinturnicidae* sp.	MK140216/*Bartonella* sp./110CJ1874312 (100%)
4	YNSB/BM06	*Bartonella* sp.	YNSB/BM06	*Spinturnicidae* sp.	MT362931/*Bartonella* sp./BE-12 (99.47%)
5	YNMS/BT01	*Bartonella* sp.	YNMS/BT01	*Ixodes vespertilionis*	KX655829/*Bartonella* sp./SD-70/2015 (98.42%)
6	YNWD/BC02	*Bartonella* sp.	YNWD/BC02	*Thaumapsylla* sp.	KM215691/*Bartonella* chomelii/Ru55 (88.42%)
7	YNWD/BC03	*Bartonella* sp.	YNWD/BC03	*Thaumapsylla* sp.	FJ589054/*Bartonella* sp./RT230YN (94.74%)
8	YNBS/BF03	*Bartonella* sp.	YNBS/BF03	*Eucampsipoda africana*	MZ388461/*Bartonella* sp./UM1a (95.26%)
9	YNBS/BF04	*Bartonella* sp.	YNBS/BF04	*Eucampsipoda africana*	KM030506/*Bartonella* sp./B23975 (93.42%)
10	YNBS/BF06	*Bartonella* sp.	YNBS/BF06	*Eucampsipoda africana*	MZ388461/*Bartonella* sp./UM1a (94.99%)
11	YNML1/BF11	*Bartonella* sp.	YNML1/BF11	*Eucampsipoda sundaica*	KM030526/*Bartonella* sp./B40908 (93.40%)
12	YNML1/BF12	*Bartonella* sp.	YNML1/BF12	*Eucampsipoda sundaica*	KP010193/*Bartonella* sp./KEL17 (93.16%)
13	YNML2/BF13	*Bartonella* sp.	YNML2/BF13	*Phthiridium* sp.	KP100360/*Bartonella* sp./B110 (95.26%)
14	YNMJ/BF15	*Bartonella* sp.	YNMJ/BF15	*Nycteribiidae* sp.	KM030503/*Bartonella* sp./B23797 (97.89%)
15	YNMJ/BF16	*Bartonella* sp.	YNMJ/BF16	*Nycteribiidae* sp.	MT362931/*Bartonella* sp./BE-12 (98.94%)
16	YNTC/BF17	*Bartonella* sp.	YNTC/BF17	*Nycteribia* sp.	KX655829/*Bartonella* sp./SD-70/2015 (98.68%)
17	YNWD/BF23	*Bartonella* sp.	YNWD/BF23	*Eucampsipoda africana*	KM030506/*Bartonella* sp./B23975 (93.42%)
18	YNXY/BF24	*Bartonella* sp.	YNXY/BF24	*Penicillidia monoceros*	KX655829/*Bartonella* sp./SD-70/2015 (99.47%)
19	YNXY/BF27	*Bartonella* sp.	YNXY/BF27	*Nycteribia* sp.	KX655829/*Bartonella* sp./SD-70/2015 (94.99%)
20	YNYD/BF28	*Bartonella* sp.	YNYD/BF28	*Phthiridium* sp.	KP100348/*Bartonella* sp./B056 (95.79%)

## Data Availability

Sequences obtained in the current study were submitted to GenBank with accession numbers OP433671-OP433727.

## Supplementary Materials

The following supporting information can be downloaded at https://www.mdpi.com/article/10.3390/pathogens11111283/s1, Table S1: Summary of sample mixed pools and the result of *Bartonella* detection. Table S2: GenBank accession and information of reference sequences for *gltA*. Table S3: GenBank accession and information of reference sequences for MLSA. Table S4: Primers used in the current study. Table S5: Sample distribution of 10 trapping sites. Figure S1: Maximum likelihood tree for 40 mixed pools of bat ectoparasites based on the partial nucleotide sequence of the *COI* gene.

## References

[1] Dvm E.B.B., Maggi R., Chomel B.B., Lappin M.R. (2010). Bartonellosis: An emerging infectious disease of zoonotic importance to animals and human beings. J. Veter. Emerg. Crit. Care.

[2] Harms A., Dehio C. (2012). Intruders below the Radar: Molecular Pathogenesis of *Bartonella* spp.. Clin. Microbiol. Rev..

[3] Okaro U., Addisu A., Casanas B., Anderson B. (2017). *Bartonella* Species, an Emerging Cause of Blood-Culture-Negative Endocarditis. Clin. Microbiol. Rev..

[4] Rao H., Li S., Lu L., Wang R., Song X., Sun K., Shi Y., Li D., Yu J. (2021). Genetic diversity of *Bartonella* species in small mammals in the Qaidam Basin, western China. Sci. Rep..

[5] Lin E.Y., Tsigrelis C., Baddour L.M., Lepidi H., Rolain J.-M., Patel R., Raoult D. (2010). *Candidatus Bartonella mayotimonensis* and Endocarditis. Emerg. Infect. Dis..

[6] Kosoy M., McKee C., Albayrak L., Fofanov Y. (2018). Genotyping of *Bartonella* bacteria and their animal hosts: Current status and perspectives. Parasitology.

[7] Calisher C.H., Childs J.E., Field H.E., Holmes K.V., Schountz T. (2006). Bats: Important Reservoir Hosts of Emerging Viruses. Clin. Microbiol. Rev..

[8] Banerjee A., Baker M.L., Kulcsar K., Misra V., Plowright R., Mossman K. (2020). Novel Insights into Immune Systems of Bats. Front. Immunol..

[9] Dacheux L., Cervantes-Gonzalez M., Guigon G., Thiberge J.-M., Vandenbogaert M., Maufrais C., Caro V., Bourhy H. (2014). A Preliminary Study of Viral Metagenomics of French Bat Species in Contact with Humans: Identification of New Mammalian Viruses. PLoS ONE.

[10] Kading R.C., Schountz T. (2016). Flavivirus Infections of Bats: Potential Role in Zika Virus Ecology. Am. J. Trop. Med. Hyg..

[11] Afelt A., Lacroix A., Zawadzka-Pawlewska U., Pokojski W., Buchy P., Frutos R. (2017). Distribution of bat-borne viruses and environment patterns. Infect. Genet. Evol..

[12] Mühldorfer K., Speck S., Wibbelt G. (2011). Diseases in free-ranging bats from Germany. BMC Veter. Res..

[13] Bai Y., Urushadze L., Osikowicz L., McKee C., Kuzmin I., Kandaurov A., Babuadze G., Natradze I., Imnadze P., Kosoy M. (2017). Molecular Survey of Bacterial Zoonotic Agents in Bats from the Country of Georgia (Caucasus). PLoS ONE.

[14] Corduneanu A., Sándor A.D., Ionică A.M., Hornok S., Leitner N., Bagó Z., Stefke K., Fuehrer H.-P., Mihalca A.D. (2018). *Bartonella* DNA in heart tissues of bats in central and eastern Europe and a review of phylogenetic relations of bat-associated bartonellae. Parasites Vectors.

[15] Nabeshima K., Sato S., Kabeya H., Kato C., Suzuki K., Maruyama S. (2020). Isolation and genetic properties of *Bartonella* in eastern bent-wing bats (*Miniopterus fuliginosus*) in Japan. Infect. Genet. Evol..

[16] Han H., Li Z., Li X., Liu J., Peng Q., Wang R., Gu X., Jiang Y., Zhou C., Li D. (2022). Bats and their ectoparasites (*Nycteribiidae* and *Spinturnicidae*) carry diverse novel *Bartonella* genotypes, China. Transbound. Emerg. Dis..

[17] Poofery J., Narapakdeesakul D., Riana E., Arnuphapprasert A., Nugraheni Y.R., Ngamprasertwong T., Wangthongchaicharoen M., Soisook P., Bhodhibundit P., Kaewthamasorn M. (2022). Molecular identification and genetic diversity of *Bartonella* spp. in 24 bat species from Thailand. Transbound. Emerg. Dis..

[18] Bai Y., Osinubi M.O.V., Osikowicz L., McKee C., Vora N.M., Rizzo M.R., Recuenco S., Davis L., Niezgoda M., Ehimiyein A.M. (2018). Human Exposure to Novel *Bartonella* Species from Contact with Fruit Bats. Emerg. Infect. Dis..

[19] McKee C.D., Bai Y., Webb C.T., Kosoy M.Y. (2021). Bats are key hosts in the radiation of mammal-associated *Bartonella* bacteria. Infect. Genet. Evol..

[20] Breitschwerdt E.B., Kordick D.L. (2000). *Bartonella* infection in animals: Carriership, reservoir potential, pathogenicity, and zoonotic potential for human infection. Clin. Microbiol. Rev..

[21] Billeter S.A., Levy M.G., Chomel B.B., Breitschwerdt E.B. (2008). Vector transmission of *Bartonella* species with emphasis on the potential for tick transmission. Med. Veter. Èntomol..

[22] Reis C., Cote M., Le Rhun D., Lecuelle B., Levin M.L., Vayssier-Taussat M., Bonnet S.I. (2011). Vector Competence of the Tick *Ixodes ricinus* for Transmission of *Bartonella* birtlesii. PLOS Negl. Trop. Dis..

[23] Wechtaisong W., Bonnet S.I., Lien Y.-Y., Chuang S.-T., Tsai Y.-L. (2020). Transmission of *Bartonella henselae* within *Rhipicephalus sanguineus*: Data on the Potential Vector Role of the Tick. PLOS Negl. Trop. Dis..

[24] Szentiványi T., Christe P., Glaizot O. (2019). Bat Flies and Their Microparasites: Current Knowledge and Distribution. Front. Veter. Sci..

[25] Xu Z., Feng Y., Chen X., Shi M., Fu S., Yang W., Liu W.J., Gao G.F., Liang G. (2022). Virome of Bat-Infesting Arthropods: Highly Divergent Viruses in Different Vectors. J. Virol..

[26] Stuckey M.J., Chomel B.B., de Fleurieu E.C., Aguilar-Setién A., Boulouis H.J., Chang C.C. (2017). *Bartonella*, bats and bugs: A review. Comp. Immunol. Microbiol. Infect. Dis..

[27] Morse S.F., Olival K.J., Kosoy M., Billeter S., Patterson B.D., Dick C.W., Dittmar K. (2012). Global distribution and genetic diversity of *Bartonella* in bat flies (Hippoboscoidea, Streblidae, Nycteribiidae). Infect. Genet. Evol..

[28] Reeves W.K., Loftis A.D., Gore J.A., Dasch G.A. (2005). Molecular evidence for novel *Bartonella* species in *Trichobius major* (Diptera: Streblidae) and *Cimex adjunctus* (Hemiptera: Cimicidae) from two southeastern bat caves, U.S.A. J. Vector Ecol..

[29] Billeter S.A., Hayman D.T., Peel A.J., Baker K., Wood J.L., Cunningham A., Suu-Ire R., Dittmar K., Kosoy M.Y. (2012). *Bartonella* species in bat flies (Diptera: Nycteribiidae) from western Africa. Parasitology.

[30] Brook C.E., Bai Y., Dobson A.P., Osikowicz L.M., Ranaivoson H.C., Zhu Q., Kosoy M.Y., Dittmar K. (2015). *Bartonella* spp. in Fruit Bats and Blood-Feeding Ectoparasites in Madagascar. PLOS Negl. Trop. Dis..

[31] Qiu Y., Kajihara M., Nakao R., Mulenga E., Harima H., Hang’Ombe B.M., Eto Y., Changula K., Mwizabi D., Sawa H. (2020). Isolation of *Candidatus Bartonella rousetti* and Other Bat-associated *Bartonellae* from Bats and Their Flies in Zambia. Pathogens.

[32] Lee H., Seo M.-G., Lee S.-H., Oem J.-K., Kim S.-H., Jeong H., Kim Y., Jheong W.-H., Kwon O.-D., Kwak D. (2021). Relationship among bats, parasitic bat flies, and associated pathogens in Korea. Parasites Vectors.

[33] Low V.L., Tan T.K., Tohiran K.A., Lim Y.A.L., AbuBakar S., Nasir D.M. (2022). A novel clade of bat-associated *Bartonella* detected in the bat fly *Leptocyclopodia ferrari* (Diptera: Nycteribiidae) parasitizing *Cynopterus brachyotis* (Chiroptera: Pteropodidae). Veter. Microbiol..

[34] Szubert-Kruszyńska A., Stańczak J., Cieniuch S., Podsiadły E., Postawa T., Michalik J. (2019). *Bartonella* and *Rickettsia* Infections in Haematophagous *Spinturnix myoti* Mites (Acari: Mesostigmata) and their Bat Host, *Myotis myotis* (Yangochiroptera: Vespertilionidae), from Poland. Microb. Ecol..

[35] Tahir D., Socolovschi C., Marié J.-L., Ganay G., Berenger J.-M., Bompar J.-M., Blanchet D., Cheuret M., Mediannikov O., Raoult D. (2016). New Rickettsia species in soft ticks *Ornithodoros hasei* collected from bats in French Guiana. Ticks Tick-Borne Dis..

[36] Hornok S., Szőke K., Meli M.L., Sándor A.D., Görföl T., Estók P., Wang Y., Tu V.T., Kováts D., Boldogh S.A. (2019). Molecular detection of vector-borne bacteria in bat ticks (Acari: Ixodidae, Argasidae) from eight countries of the Old and New Worlds. Parasites Vectors.

[37] Reeves W.K., Rogers T.E., Durden L.A., Dasch G. (2007). Association of *Bartonella* with the fleas (Siphonaptera) of rodents and bats using molecular techniques. J. Vector Ecol..

[38] Veikkolainen V., Vesterinen E.J., Lilley T.M., Pulliainen A.T. (2014). Bats as Reservoir Hosts of Human Bacterial Pathogen, *Bartonella mayotimonensis*. Emerg. Infect. Dis..

[39] Bush S.E., Robbins R.G. (2012). New host and locality records for *Ixodes simplex* Neumann and *Ixodes vespertilionis* Koch (Acari: Ixodidae) from bats (Chiroptera: Hipposideridae, Rhinolophidae and Vespertilionidae) in southern China. Int. J. Acarol..

[40] Hornok S., Görföl T., Estók P., Tu V.T., Kontschán J. (2016). Description of a new tick species, *Ixodes collaris* n. sp. (Acari: Ixodidae), from bats (Chiroptera: Hipposideridae, Rhinolophidae) in Vietnam. Parasites Vectors.

[41] Lin J.-W., Hsu Y.-M., Chomel B.B., Lin L.-K., Pei J.-C., Wu S.-H., Chang C.-C. (2012). Identification of novel *Bartonella* spp. in bats and evidence of Asian gray shrew as a new potential reservoir of *Bartonella*. Veter. Microbiol..

[42] Han H.-J., Wen H.-L., Zhao L., Liu J.-W., Luo L.-M., Zhou C.-M., Qin X.-R., Zhu Y.-L., Zheng X.-X., Yu X.-J. (2017). Novel *Bartonella* Species in Insectivorous Bats, Northern China. PLoS ONE.

[43] Birtles R., Raoult D. (1996). Comparison of Partial Citrate Synthase Gene (*gltA*) Sequences for Phylogenetic Analysis of *Bartonella* Species. Int. J. Syst. Bacteriol..

[44] La Scola B., Zeaiter Z., Khamis A., Raoult D. (2003). Gene-sequence-based criteria for species definition in bacteriology: The *Bartonella* paradigm. Trends Microbiol..

[45] Vayssier-Taussat M., Le Rhun D., Bonnet S., Cotté V. (2009). Insights in *Bartonella* Host Specificity. Ann. N. Y. Acad. Sci..

[46] Lei B.R., Olival K.J. (2014). Contrasting Patterns in Mammal–Bacteria Coevolution: *Bartonella* and *Leptospira* in Bats and Rodents. PLOS Negl. Trop. Dis..

[47] Dick C.W. (2007). High host specificity of obligate ectoparasites. Ecol. Èntomol..

[48] Kumar S., Stecher G., Suleski M., Hedges S.B. (2017). TimeTree: A Resource for Timelines, Timetrees, and Divergence Times. Mol. Biol. Evol..

[49] Bininda-Emonds O.R.P., Cardillo M., Jones K.E., MacPhee R.D.E., Beck R.M.D., Grenyer R., Price S.A., Vos R.A., Gittleman J.L., Purvis A. (2007). The delayed rise of present-day mammals. Nature.

[50] Feng Y., Li Y., Fu S., Li X., Song J., Zhang H., Yang W., Zhang Y., Pan H., Liang G. (2017). Isolation of Kaeng Khoi virus (KKV) from *Eucampsipoda sundaica* bat flies in China. Virus Res..

[51] Folmer O., Black M., Hoeh W., Lutz R., Vrijenhoek R. (1994). DNA primers for amplification of mitochondrial cytochrome c oxidase subunit I from diverse metazoan invertebrates. Mol. Mar. Biol. Biotechnol..

[52] Norman A.F., Regnery R., Jameson P., Greene C., Krause D.C. (1995). Differentiation of *Bartonella*-like isolates at the species level by PCR-restriction fragment length polymorphism in the citrate synthase gene. J. Clin. Microbiol..

[53] Bai Y., Hayman D.T.S., McKee C., Kosoy M.Y. (2015). Classification of *Bartonella* Strains Associated with Straw-Colored Fruit Bats (*Eidolon helvum*) across Africa Using a Multi-locus Sequence Typing Platform. PLOS Negl. Trop. Dis..

[54] de Oliveira J.G., Rozental T., Guterres A., Teixeira B.R., Andrade-Silva B.E., da Costa-Neto S.F., Furtado M.C., Moratelli R., D’Andrea P.S., Lemos E.R.S. (2020). Investigation of *Bartonella* spp. in brazilian mammals with emphasis on rodents and bats from the Atlantic Forest. Int. J. Parasitol. Parasites Wildl..

[55] Roux V., Raoult D. (1995). The 16S-23S rRNA intergenic spacer region of *Bartonella* (Rochalimaea) species is longer than usually described in other bacteria. Gene.

[56] Paziewska A., Harris P.D., Zwolińska L., Bajer A., Siński E. (2010). Recombination Within and Between Species of the Alpha Proteobacterium *Bartonella* Infecting Rodents. Microb. Ecol..

[57] Katoh K., Standley D.M. (2013). MAFFT Multiple Sequence Alignment Software Version 7: Improvements in Performance and Usability. Mol. Biol. Evol..

[58] Capella-Gutiérrez S., Silla-Martínez J.M., Gabaldón T. (2009). trimAl: A tool for automated alignment trimming in large-scale phylogenetic analyses. Bioinformatics.

[59] Guindon S., Dufayard J.-F., Lefort V., Anisimova M., Hordijk W., Gascuel O. (2010). New Algorithms and Methods to Estimate Maximum-Likelihood Phylogenies: Assessing the Performance of PhyML 3.0. Syst. Biol..

