# Supplementary material for: Molecular Detection and Phylogenetic Analyses of Diverse Bartonella Species in Bat Ectoparasites Collected from Yunnan Province, China
Source: Pathogens. 2022 Nov 1;11(11):1283. doi: 10.3390/pathogens11111283 (PMC9699461; doi:10.3390/pathogens11111283)
Supplement: Supplementary file 1 [file pathogens-11-01283-s001.zip › pathogens-1972595-supplementary.pdf]

Table S1. Summary of sample mixed pools and result of *Bartonella* detection

order	POOL NO.	HOST FAMILY	HOST SPECIES	NUM	LOCATION	DATE	PCR			
							gltA	*ftsZ*	*rpoB*	ITS
1	YNML1/BM01	*Spinturnicidae*	*Spinturnicidae* sp.	16	Mengla	2012				
2	YNML1/BM02	*Spinturnicidae*	*Spinturnicidae* sp.	17	Mengla	2012	+	+	+	+
3	YNML2/BM03	*Spinturnicidae*	*Spinturnicidae* sp.	30	Menglian	2012	+	+	+	+
4	YNML2/BM04	*Spinturnicidae*	*Spinturnicidae* sp.	25	Menglian	2012	+	+	+	-
5	YNMJ/BM05	*Spinturnicidae*	*Spinturnicidae* sp.	15	Mojiang	2013				
6	YNSB/BM06	*Spinturnicidae*	*Spinturnicidae* sp.	10	Shuangbai	2013	+	+	-	+
7	YNMS/BT01	*Ixodidae*	*Ixodes vespertilionis*	1	Mangshi	2012	+	+	+	+
8	YNMJ/BT02	*Ixodidae*	*Ixodes collaris*	2	Mojiang	2013				
9	YNTC/BT03	*Ixodidae*	*Ixodes collaris*	2	Tengchong	2012				
10	YNBS/BC01	*Ischnopsyllidae*	*Thaumapsylla* sp.	1	Baoshan	2014				
11	YNWD/BC02	*Ischnopsyllidae*	*Thaumapsylla* sp.	4	Wanding	2013	+	+	+	-
12	YNWD/BC03	*Ischnopsyllidae*	*Thaumapsylla* sp.	16	Wanding	2014	+	+	+	+
13	YNBS/BF01	*Nycteribiidae*	*Eucampsipoda africana*	19	Baoshan	2014				
14	YNBS/BF02	*Nycteribiidae*	*Eucampsipoda africana*	17	Baoshan	2014				
15	YNBS/BF03	*Nycteribiidae*	*Eucampsipoda africana*	13	Baoshan	2014	+	+	+	+
16	YNBS/BF04	*Nycteribiidae*	*Eucampsipoda africana*	10	Baoshan	2014	+	+	+	+
17	YNBS/BF05	*Nycteribiidae*	*Eucampsipoda africana*	6	Baoshan	2014				
18	YNBS/BF06	*Nycteribiidae*	*Eucampsipoda africana*	14	Baoshan	2014	+	+	+	+
19	YNBS/BF07	*Streblidae*	*Brachytarsina* sp.	1	Baoshan	2014				
20	YNMS/BF08	*Nycteribiidae*	*Nycteribiidae* sp.	9	Mangshi	2012				
21	YNMS/BF09	*Nycteribiidae*	*Nycteribiidae* sp.	5	Mangshi	2012				
22	YNMS/BF10	*Nycteribiidae*	*Nycteribiidae* sp.	5	Mangshi	2012				
23	YNML1/BF11	*Nycteribiidae*	*Eucampsipoda sundaica*	19	Mengla	2012	+	+	+	+
24	YNML1/BF12	*Nycteribiidae*	*Eucampsipoda sundaica*	24	Mengla	2012	+	+	+	+
25	YNML2/BF13	*Nycteribiidae*	*Phthiridium* sp.	4	Menglian	2014	+	+	+	+
26	YNMJ/BF14	*Streblidae*	*Brachytarsina kanoi*	8	Mojiang	2013				
27	YNMJ/BF15	*Nycteribiidae*	*Nycteribiidae* sp.	12	Mojiang	2013	+	+	+	+
28	YNMJ/BF16	*Nycteribiidae*	*Nycteribiidae* sp.	14	Mojiang	2013	+	+	-	+
29	YNTC/BF18	*Nycteribiidae*	*Phthiridium* sp.	5	Tengchong	2012				
30	YNTC/BF17	*Nycteribiidae*	*Nycteribia* sp.	10	Tengchong	2012	+	+	-	+
31	YNWD/BF19	*Nycteribiidae*	*Nycteribia* sp.	13	Wanding	2012				
32	YNWD/BF20	*Nycteribiidae*	*Eucampsipoda inermis*	8	Wanding	2013				
33	YNWD/BF21	*Streblidae*	*Brachytarsina* sp.	2	Wanding	2013				
34	YNWD/BF22	*Nycteribiidae*	*Eucampsipoda africana*	12	Wanding	2014				
35	YNWD/BF23	*Nycteribiidae*	*Eucampsipoda africana*	11	Wanding	2014	+	+	+	+
36	YNXY/BF24	*Nycteribiidae*	*Penicillidia monoceros*	8	Xiangyun	2013	+	+	+	+
37	YNXY/BF25	*Nycteribiidae*	*Nycteribia* sp.	8	Xiangyun	2014				
38	YNXY/BF26	*Nycteribiidae*	*Nycteribia* sp.	10	Xiangyun	2014				
39	YNXY/BF27	*Nycteribiidae*	*Nycteribia* sp.	18	Xiangyun	2014	+	+	+	+
40	YNYD/BF28	*Nycteribiidae*	*Phthiridium* sp.	10	Yongde	2014	+	+	+	+

Note: "+" indicated pools that positive to *Bartonella* detection, while "-" and cell blank indicated negative.

Table S2. GenBank accession and information of reference sequences for *gltA*

gltA accession	Classification	Strain	Country	Host species
MK140216	*Bartonella* sp.	110CJ1874312	Romania	*Miniopterus schreibersii*
KF003115	*Bartonella* sp.	1157-3	Finland	*Eptesicus nilssoni*
KF003122	*Bartonella* sp.	1160-1	Finland	*Myotis daubentonii*
KF003129	*Bartonella* sp.	2574-1	Finland	*Myotis daubentonii*
KY629840	*Bartonella* sp.	A14	Mexico	*Desmodus rotundus*
KY629868	*Bartonella* sp.	A3	Mexico	*Desmodus rotundus*
KY629834	*Bartonella* sp.	A5	Mexico	*Desmodus rotundus*
KP100341	*Bartonella* sp.	B005	Vietnam	*Megaderma spasma*
KP100348	*Bartonella* sp.	B056	Vietnam	*Rhinolophus acuminatus*
KP100360	*Bartonella* sp.	B110	Vietnam	*Hipposideros larvatus*
KM030506	*Bartonella* sp.	B23975	Kenya	*Eidolon helvum*
HM597187	*Bartonella* sp.	B29042	Guatemala	*Desmodus rotundus*
JQ071382	*Bartonella* sp.	B32954	Peru	*Artibeus planirostris*
KM030518	*Bartonella* sp.	B40005	Cameroon	*Eidolon helvum*
MK140370	*Bartonella* sp.	B44672	Georgia	*Myotis blythii*
LC483820	*Bartonella* sp.	bat2-1	Japan	*Miniopterus schreibersi fliginosus*
KJ530742	*Bartonella* sp.	Bc37076	Puerto Rico	*Brachyphylla cavernarum*
KX807178	*Bartonella* sp.	DMR02005	USA	*Myotis lucifugus*
KY629879	*Bartonella* sp.	DR13	Mexico	*Desmodus rotundus*
KY629857	*Bartonella* sp.	DR22	Mexico	*Desmodus rotundus*
MN270084	*Bartonella* sp.	E1 B23987	Kenya	*Eidolon helvum*
KM030503	*Bartonella* sp.	E2 B23979	Kenya	*Eidolon helvum*
KM030511	*Bartonella* sp.	E2 B24225	Kenya	*Eidolon helvum*
MN270085	*Bartonella* sp.	E2 B39294	Ghana	*Eidolon helvum*
KM030522	*Bartonella* sp.	E2 B40396	Tanzania	*Eidolon helvum*
MN270087	*Bartonella* sp.	E3 B39300	Ghana	*Eidolon helvum*
KM030516	*Bartonella* sp.	E4 B39301	Ghana	*Eidolon helvum*
KM030513	*Bartonella* sp.	E5 B39249	Ghana	*Eidolon helvum*
KM030520	*Bartonella* sp.	E5 B40014	Tanzania	*Eidolon helvum*
KM030526	*Bartonella* sp.	E5 B40908	Uganda	*Eidolon helvum*
KY629885	*Bartonella* sp.	F14	Mexico	*Pteronotus parnellii*
KY629884	*Bartonella* sp.	F2	Mexico	*Pteronotus parnellii*
HM545137	*Bartonella* sp.	H-556	Kenya	*Hipposideros vittatus*
KY629850	*Bartonella* sp.	J3	Mexico	*Sturnira lilium*
KP010193	*Bartonella* sp.	KEL17	Madagascar	*Eidolon dupreanum*
KY232214	*Bartonella* sp.	KP215	Thailand	*Hipposideros larvatus*
AJ871613	*Bartonella* sp.	M406	UK	*Myotis daubentonii*
KY629849	*Bartonella* sp.	M9	Mexico	*Desmodus rotundus*
KF418812	*Bartonella* sp.	Mi-BA38	Nigeria	*Micropteropus* sp.
KJ530743	*Bartonella* sp.	Mr37075	Puerto Rico	*Monophyllus redmani*
JF500511	*Bartonella* sp.	No.05	Taiwan	*Miniopterus fuliginosus*
JF500513	*Bartonella* sp.	No.07	Taiwan	*Miniopterus fuliginosus*

HM363764	*Bartonella* sp.	R-191	Nigeria	*Rousettus aegyptiacus*
MZ208702	*Bartonella* sp.	S833	China	*Myotis adversus*
KX655835	*Bartonella* sp.	SD117	China	*Myotis pequinius*
KX655829	*Bartonella* sp.	SD70	China	*Myotis fimbriatus*
KX655839	*Bartonella* sp.	SD78	China	*Myotis fimbriatus*
KR822802	*Bartonella hemsundetensis*	bat2053	Finland	*Myotis daubentonii*
KF003137	*Bartonella* sp.	NB-1.2	Finland	*Siphonaptera* sp.
JX416252	*Bartonella* sp.	05 01 07	Laos	*Phthiridium* sp. *scissa* group
MK140327	*Bartonella* sp.	15aCJ353606	Romania	*Penicillidia conspicua*
MK140280	*Bartonella* sp.	173CJ2734392	Romania	*Nycteribia kolenatii*
JX416251	*Bartonella* sp.	23 03 04	Panama	*Strebla diaemi*
MK140298	*Bartonella* sp.	52bCJ874156	Romania	*Nycteribia kolenatii*
LC461053	*Bartonella* sp.	Batfly-7	Zambia	*Eucampsipoda africana*
MT362931	*Bartonella* sp.	BE-12	South Korea	*Nycteribia allotopa*
MT362932	*Bartonella* sp.	BE-16	South Korea	*Penicillidia jenynsii*
MT362936	*Bartonella* sp.	BE-58	South Korea	*Phthiridium hindlei*
JN172074	*Bartonella* sp.	Cg 462	Equatorial Guinea	*Cyclopodia greefi*
JN172035	*Bartonella* sp.	Cg K1-2	Ghana	*Cyclopodia greefi*
JX416248	*Bartonella* sp.	CWD974	Mexico	*Trichobius johnsonae*
JX416253	*Bartonella* sp.	DR0583	Dominica	*Trichobius adamsi*
MZ208684	*Bartonella* sp.	FP13	China	*Nycteribiidae* spp.
MZ208687	*Bartonella* sp.	FP5-1	China	*Nycteribiidae* spp.
MZ208690	*Bartonella* sp.	FS2-2	China	*Penicillidia monoceros*
JX416239	*Bartonella* sp.	JAE1033	Philippines	*Leptocyclopodia sp.*
KP010152	*Bartonella* sp.	KEL02-3	Madagascar	*Cyclopodia dubia*
KP010155	*Bartonella* sp.	KEL04-1	Madagascar	*Cyclopodia dubia*
KP010164	*Bartonella* sp.	KEL19-1	Madagascar	*Cyclopodia dubia*
JX416238	*Bartonella* sp.	Mala15	Malaysia	*Phthiridium fraterna*
MH486969	*Bartonella* sp.	Nycteribiid	China	bat fly
JX416255	*Bartonella* sp.	P2874	Philippines	*Cyclopodia simulans*
MZ208694	*Bartonella* sp.	SFP4-2	China	*Nycteribiidae* spp.
MZ388461	*Bartonella* sp.	UM1a	Malaysia	bat fly
JX416246	Uncultured bacterium	Mala11	Malaysia	*Basilia coronata*
JX416241	Uncultured bacterium	ZAG03	Slovenia	*Basilia nattereri*
JQ695839	*Bartonella* sp.	AS025	Poland	*Spinturnix myoti*
JQ695840	*Bartonella* sp.	AS067	Poland	*Spinturnix myoti*
MZ208698	*Bartonella* sp.	MP8-2	China	bat mite
MH544201	*Bartonella* sp.	Ia23	Hungary	*Ixodes ariadnae*
MH578453	*Bartonella hemsundetensis*	Iv76	Romania	*Ixodes vespertilionis*
NC_007618	*Brucella abortus*	2308	Out group	Out group
NZ_WMBQ01000001	*Hyphomicrobium album*	XQ2	Out group	Out group

Table S3. GenBank accession and information of reference sequences for MLSA

gltA	*ftsZ*	*rpoB*	Classification	Strain	Country	Host specis	Host order
NZ_CP015625	NZ_CP015625	NZ_CP015625	*Bartonella apis*	BBC0122	Switzerland	*Apis mellifera*	*Arthropoda*
MZ208687	MZ208708	MZ208744	*Bartonella* sp.	FP5-1	China	*Nycteribiidae* spp.	*Arthropoda*
MZ208684	MZ208715	MZ208744	*Bartonella* sp.	FP13	China	*Nycteribiidae* spp.	*Arthropoda*
MZ208698	MZ208727	MZ208759	*Bartonella* sp.	MP8-2	China	*Spinturnicidae* spp.	*Arthropoda*
KF199896	KF193408	KF218220	*Bartonella bovis*	B25100	Thailand	*Bos taurus*	*Artiodactyla*
AF293392	AB290192	AB290188	*Bartonella capreoli*	IBS193	France	*Capreolus capreolus*	*Artiodactyla*
KM215690	KM215688	KM215706	*Bartonella chomelii*	Ru15	Spain	*Bos taurus*	*Artiodactyla*
KM371042	KM371051	KJ909806	*Bartonella dromedarii*	69C	Israel	*Camelus dromedarius*	*Artiodactyla*
HG977196	HG977196	HG977196	*Bartonella schoenbuchensis*	MVT06	Sweden	*Alces alces*	*Artiodactyla*
NC_014932	NC_014932	NC_014932	*Bartonella clarridgeiae*	73	France	*Felis catus*	*Carnivora*
NZ_KL407337	NZ_KL407337	NZ_KL407337	*Bartonella rochalimae*	BMGH	Peru	*Carnivora* spp.	*Carnivora*
NZ_CP019787	NZ_CP019787	NZ_CP019787	*Bartonella* sp.	JB15	Japan	*Meles anakuma*	*Carnivora*
KP715473	KP715472	KP715475	*Bartonella rolaini*	C65	French Guiana	*Noctilio albiventris*	*Chiroptera*
KF003115	KF003121	KF003118	*Bartonella* sp.	1157-3	Finland	*Eptesicus nilssoni*	*Chiroptera*
KF003129	KF003135	KF003132	*Bartonella* sp.	2574-1	Finland	*Myotis daubentonii*	*Chiroptera*
KY629840	KY629820	KY629921	*Bartonella* sp.	A14	Mexico	*Desmodus rotundus*	*Chiroptera*
KY629834	KY629824	KY629925	*Bartonella* sp.	A5	Mexico	*Desmodus rotundus*	*Chiroptera*
KY629854	KY629806	KY629907	*Bartonella* sp.	D4	Mexico	*Sturnira ludovici*	*Chiroptera*
KY629879	KY629803	KY629904	*Bartonella* sp.	DR13	Mexico	*Desmodus rotundus*	*Chiroptera*
KY629851	KY629799	KY629900	*Bartonella* sp.	DR24	Mexico	*Desmodus rotundus*	*Chiroptera*
KM030511	KJ999683	KM215195	*Bartonella* sp.	E2 B24225	Kenya	*Eidolon helvum*	*Chiroptera*
MN270085	KJ999686	MN270150	*Bartonella* sp.	E2 B39294	Ghana	*Eidolon helvum*	*Chiroptera*
KM030520	KJ999689	KM215202	*Bartonella* sp.	E5 B40014	Tanzania	*Eidolon helvum*	*Chiroptera*
KM030526	KJ999694	MN270154	*Bartonella* sp.	E5	Uganda	*Eidolon helvum*	*Chiroptera*

				B40908			
KY629884	KY629797	KY629898	*Bartonella* sp.	F2	Mexico	*Pteronotus parnellii*	*Chiroptera*
KY629882	KY629796	KY629897	*Bartonella* sp.	F9	Mexico	*Pteronotus parnellii*	*Chiroptera*
KY629850	KY629793	KY629894	*Bartonella* sp.	J3	Mexico	*Sturnira lilium*	*Chiroptera*
JF500513	JF500497	JF500545	*Bartonella* sp.	No.07	Taiwan	*Miniopterus fuliginosus*	*Chiroptera*
MZ208702	MZ208734	MZ208765	*Bartonella* sp.	S833	China	*Myotis davidii*	*Chiroptera*
HM363764	HM363769	HM363774	*Bartonella* sp.	R-191	Nigeria	*Rousettus aegyptiacus*	*Chiroptera*
GU168962	GU168958	GU168961	*Candidatus Bartonella antechini*	MU1-F19	Australia	*Antechinus flavipes*	*Dasyuromorphia*
NC_020300	NC_020300	NC_020300	*Bartonella australis*	Aust-NH1	Australia	*Macropus giganteus*	*Diprotodontia*
HQ444152	HQ444150	HQ444151	*Candidatus Bartonella woyliei*	WC1	Australia	*Bettongia penicillata*	*Diprotodontia*
HQ444165	HQ444163	HQ444164	*Candidatus Bartonella bandicootii*	BA1	Australia	*Oryctolagus cuniculus*	*Peramelemorphia*
NZ_KL503800	NZ_KL503800	NZ_KL503800	*Bartonella bacilliformis*	Cond044	Peru	*Perameles bougainville*	*Primates*
NZ_KK097685	NZ_KK097685	NZ_KK097685	*Bartonella bacilliformis*	HeidiMejia	Peru	*Homo sapiens*	*Primates*
NZ_KL503803	NZ_KL503805	NZ_KL503803	*Bartonella bacilliformis*	Ver097	Peru	*Homo sapiens*	*Primates*
CP003784	CP003784	CP003784	*Bartonella quintana*	RM-11	China	*Homo sapiens*	*Primates*
NC_005955	NC_005955	NC_005955	*Bartonella quintana*	Toulouse	France	*Macaca mulatta*	*Primates*
NZ_JH725147	NZ_JH725147	NZ_JH725147	*Bartonella tamiae*	Th239	Thailand	*Homo sapiens*	*Primates*
AB444979	AB602545	AB529942	*Bartonella acomydis*	KS2-1	Egypt	*Homo sapiens*	*Rodentia*
NZ_CM001557	NZ_CM001557	NZ_CM001557	*Bartonella birtlesii*	IBS325	UK	*Acomys russatus*	*Rodentia*
NZ_JH725033	NZ_JH725034	NZ_JH725033	*Bartonella elizabethae*	F9251	USA	*Apodemus sylvaticus*	*Rodentia*
NC_012846	NC_012846	NC_012846	*Bartonella grahamii*	as4aup	Sweden	*Rattus, Bandicota spp.*	*Rodentia*
KY555066	KY555065	KY555068	*Bartonella mastomydis*	008	Senegal	*Apodemus sylvaticus*	*Rodentia*
AB444978	AB602543	AB602555	*Bartonella pachyuromydis*	FN15-2	Netherlands	*Mastomys erythroleucus*	*Rodentia*
NZ_WMBQ01000001	NZ_WMBQ01000001	NZ_WMBQ01000001	*Hyphomicrobium album*	XQ2	Outgroup	Outgroup	Outgroup

Table S4. Primers used in the current study

Target species	gene	Primers	Sequences (5′-3′)	PCR products (bp)	Reference
Bat ectoparasites	*COI*	LCO1490	GGTCAACAAATCATAAAGATATTGG	710	[1]
		HCO2198	TAAACTTCAGGGTGACCAAAAAATCA		
Bartonella bacteria	*gltA*	*gltA*-F	GGGGACCAGCTCATGGTGG	380	[2]
		gltA-R	AATGCAAAAAGAACAGTAAACA		
	rpoB	*rpoB*-F	CGCATTGGCTTACTTCGTATG	851	[3]
		rpoB-R	GTAGACTGATTAGAACGCTG		
	ftsZ	*ftsZ*-F	ATTAATCTGCAYCGGCCAGATAT	786	[4]
		ftsZ-R	TCATCAATRGCVCCAAARAT		
	ITS	ITS-F	AGAGGCAGGCAACCACGGTA	1211-1529	[5]
		ITS-R	GCCAAGGCATCCACC		

References

1. Folmer, O.; Black, M.; Hoeh, W.; Lutz, R.; Vrijenhoek, R. DNA primers for amplification of mitochondrial cytochrome c oxidase subunit I from diverse metazoan invertebrates. *Mol. Mar. Biol. Biotechnol.* **1994**, *3*, 294–299.
2. Norman, A.F.; Regnery, R.; Jameson, P.; Greene, C.; Krause, D.C. Differentiation of *Bartonella*-like isolates at the species level by PCR-restriction fragment length polymorphism in the citrate synthase gene. *J. Clin. Microbiol.* **1995**, *33*, 1797–1803. https://doi.org/10.1128/jcm.33.7.1797-1803.1995.
3. .Bai, Y.; Hayman, D.T.S.; McKee, C.; Kosoy, M.Y. Classification of *Bartonella* Strains Associated with Straw-Colored Fruit Bats (*Eidolon helvum*) across Africa Using a Multi-locus Sequence Typing Platform. *PLOS Neglected Trop. Dis.* **2015**, *9*, e0003478–e0003478. https://doi.org/10.1371/journal.pntd.0003478.
4. Oliveira, J.G.; Rozental, T.; Guterres, A.; Teixeira, B.R.; Andrade-Silva, B.E.; da Costa-Neto, S.F.; Furtado, M.C.; Moratelli, R.; D'Andrea, P.S.; Lemos, E.R.S. Investigation of *Bartonella* spp. in brazilian mammals with emphasis on rodents and bats from the Atlantic Forest. *Int. J. Parasitol. Parasites Wildl.* **2020**, *13*, 80–89. https://doi.org/10.1016/j.ijppaw.2020.07.004.
5. Roux, V.; Raoult, D. The 16S-23S rRNA intergenic spacer region of *Bartonella* (Rochalimaea) species is longer than usually described in other bacteria. *Gene* **1995**, *156*, 107–111. https://doi.org/10.1016/0378-1119(94)00919-j.

Table S5 Samples distribution of 10 trapping sites

NO.	Counties	Bat ectoparasites					Total
		Bat flies		Mites	Ticks	Fleas	
		Nycteribiidae	*Streblidae*				
1	Baoshan	79	1	0	0	1	81
2	Mangshi	13	6	0	1	0	20
3	Mengla	43	0	33	0	0	76
4	Menglian	4	0	55	0	0	59
5	Mojiang	26	8	15	2	0	51
6	Shangbai	0	0	10	0	0	10
7	Tengchong	15	0	0	2	0	17
8	Wanding	44	2	0	0	20	66
9	Xiangyun	44	0	0	0	0	44
10	Yongde	10	0	0	0	0	10

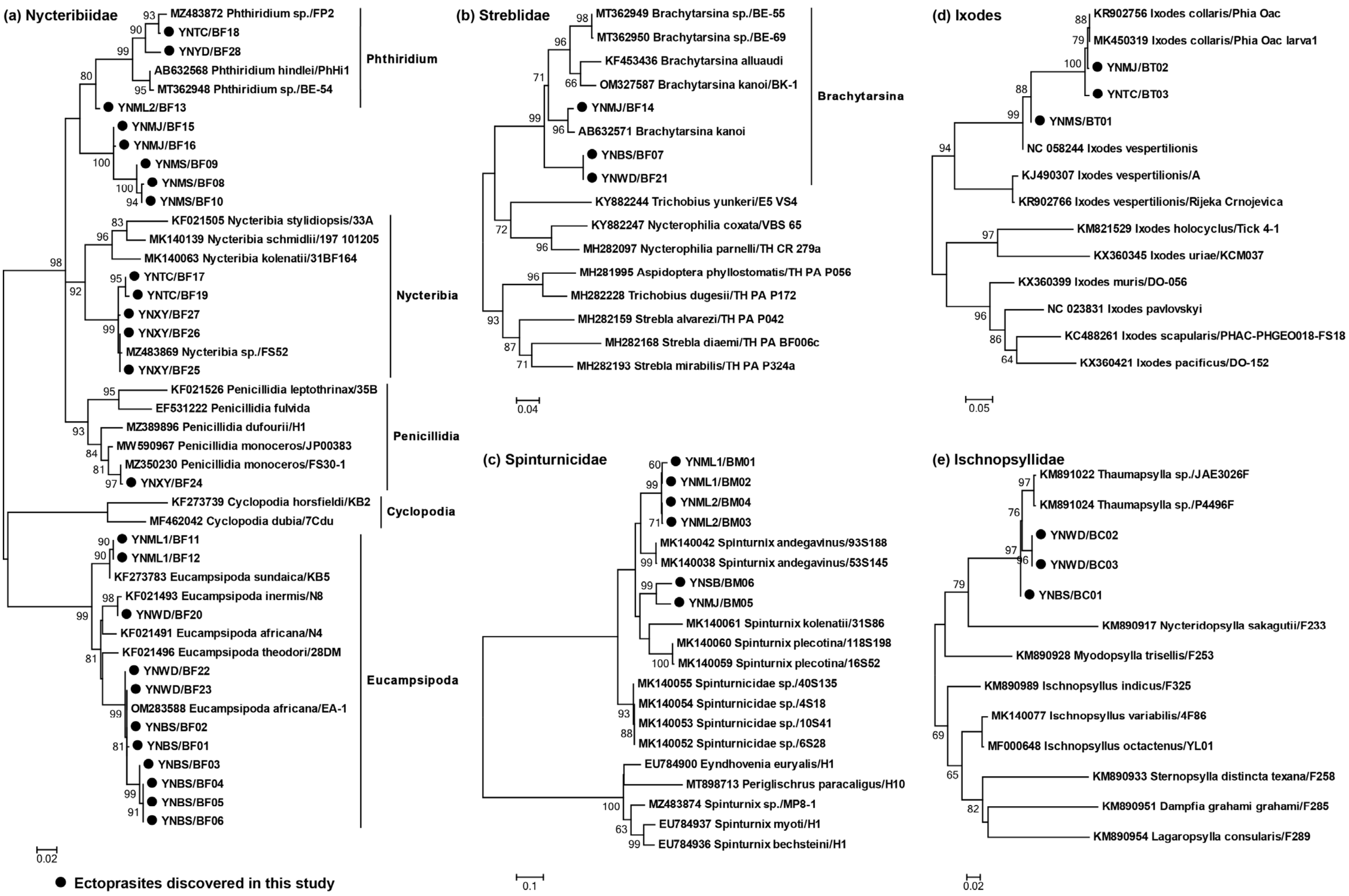

Figure S1. Maximum likelihood tree for 40 mixed pools of bat ectoparasites based on the partial nucleotide sequence of the *COI* gene. Five families of bat-specific ectoparasites were contained: (a) *Nycteribiidae*, (b) *Streblidae*, (c) *Spinturnicidae*, (d) *Ixodidae: Ixodes*, (e) *Ischnopsyllidae.* The ectoparasites newly discovered in the current study are marked with solid black circles within the phylogenetic tree.
